# Reddit and Google Activity Related to Non-COVID Epidemic Diseases Surged at Start of COVID-19 Pandemic: Retrospective Study

**DOI:** 10.2196/44603

**Published:** 2023-07-06

**Authors:** Jack A Cummins, Adam D Lipworth

**Affiliations:** 1 Manchester Essex Regional High School Manchester-by-the-Sea, MA United States; 2 Lahey Dermatology Burlington, MA United States

**Keywords:** COVID-19, Reddit, Google Trends, chikungunya, Ebola, H1N1, Middle Eastern respiratory syndrome, MERS, severe acute respiratory syndrome, SARS, Zika, infectious disease, social media, search data, search query, web-based search, information behavior, information seeking, public interest

## Abstract

**Background:**

Resources such as Google Trends and Reddit provide opportunities to gauge real-time popular interest in public health issues. Despite the potential for these publicly available and free resources to help optimize public health campaigns, use for this purpose has been limited.

**Objective:**

The purpose of this study is to determine whether early public awareness of COVID-19 correlated with elevated public interest in other infectious diseases of public health importance.

**Methods:**

Google Trends search data and Reddit comment data were analyzed from 2018 through 2020 for the frequency of keywords “chikungunya,” “Ebola,” “H1N1,” “MERS,” “SARS,” and “Zika,” 6 highly publicized epidemic diseases in recent decades. After collecting Google Trends relative popularity scores for each of these 6 terms, unpaired 2-tailed *t* tests were used to compare the 2020 weekly scores for each term to their average level over the 3-year study period. The number of Reddit comments per month with each of these 6 terms was collected and then adjusted for the total estimated Reddit monthly comment volume to derive a measure of relative use, analogous to the Google Trends popularity score. The relative monthly incidence of comments with each search term was then compared to the corresponding search term’s pre-COVID monthly comment data, again using unpaired 2-tailed *t* tests. *P* value cutoffs for statistical significance were determined a priori with a Bonferroni correction.

**Results:**

Google Trends and Reddit data both demonstrate large and statistically significant increases in the usage of each evaluated disease term through at least the initial months of the pandemic. Google searches and Reddit comments that included any of the evaluated infectious disease search terms rose significantly in the first months of 2020 above their baseline usage, peaking in March 2020. Google searches for “SARS” and “MERS” remained elevated for the entirety of the 2020 calendar year, as did Reddit comments with the words “Ebola,” “H1N1,” “MERS,” and “SARS” (*P*<.001, for each weekly or monthly comparison, respectively).

**Conclusions:**

Google Trends and Reddit can readily be used to evaluate real-time general interest levels in public health–related topics, providing a tool to better time and direct public health initiatives that require a receptive target audience. The start of the COVID-19 pandemic correlated with increased public interest in other epidemic infectious diseases. We have demonstrated that for 6 distinct infectious causes of epidemics over the last 2 decades, public interest rose substantially and rapidly with the outbreak of COVID-19. Our data suggests that for at least several months after the initial outbreak, the public may have been particularly receptive to dialogue on these topics. Public health officials should consider using Google Trends and social media data to identify patterns of engagement with public health topics in real time and to optimize the timing of public health campaigns.

## Introduction

The sluggish uptake of COVID-19 vaccines in 2021 underscores the myriad challenges public health officials face when trying to influence health-related behaviors. Recognizing both the importance and difficulty of effective public health messaging, leaders in the public health space have created tools such as the CDCynergy Social Marketing tool (US Centers for Disease Control and Prevention) to potentiate the impact of public health campaigns [1]. These tools and others seek to identify and characterize target audiences across key demographics and then reach them when they are most interested and engaged. Surveillance of digital data has been used to identify geographic areas where public health messaging may be most impactful [2,3]. Reaching the target audience at times of high interest is also important, because when public interest is high, public health campaigns are likely to be more effective [4]. However, the public interest is hard to predict, identify, or manufacture quickly enough for many public health campaigns to capture peak engagement within their target audience.

A reliable pattern of public engagement with a health issue, especially in combination with an effective tool to measure the public’s interest in real time, would help public health organizations to better schedule their outreach efforts at times when the public will be most receptive to those efforts. Public data sets such as Google Trends (Google LLC) and Reddit (Reddit Inc) provide a means for such a dynamic measurement of public interest. Google Trends is a free, open-source tool enabling the assessment over time of a search term’s relative search volume on the Google search engine, which accounts for nearly 90% of search traffic worldwide [5]. Reddit is a forum-based social media website in which users post in over 100,000 communities called “subreddits.” It is the 19th most visited website globally [6], with over 50,000,000 daily users [7].

Reddit content has been evaluated for public health purposes infrequently relative to its popularity and use, with fewer than 400 articles returning on a PubMed search of the term “Reddit” [8]. Google Trends has been used more frequently for public health purposes, including evaluating the effectiveness of public health campaigns post hoc [9-14]. However, we are not aware of efforts to use either the website or any other publicly available big data tool to time the initiation of public health efforts.

We hypothesized that high public interest in COVID-19 may correlate with a more general public interest in non-COVID infectious diseases with epidemic potential. In the event that public interest does spread from an active epidemic to more quiescent epidemic diseases that pose persistent public health threats, future epidemics might provide windows of opportunity to advance certain public health initiatives around diseases for which public interest has waned. Furthermore, such a correlation does not need to achieve extremely high generalizability or reliability to prove useful. Public data sets provide such ready access to real-time assessments of public interest, so the expectation of increased interest only needs to be high enough to merit a simple query using these free, accessible, and powerful digital tools.

This study retrospectively uses large public data sets, such as Google Trends and Reddit, to estimate the level of public interest in 6 non-COVID epidemic diseases (chikungunya, Ebola, H1N1 influenza, Middle Eastern respiratory syndrome [MERS], severe acute respiratory syndrome [SARS], and Zika virus) from 2018 through the end of 2020. This period includes approximately 2 years prior to and 1 year following the first public reports of COVID-19, enabling an evaluation of the correlation between public attention to COVID-19 and public interest in historical epidemic diseases that pose future recrudescent threats.

## Methods

### Overview

All Reddit data were retrieved using the PushShift API (Jason Baumgartner) [15]. The PushShift API was used to search through all Reddit comments from January 1, 2018, to December 31, 2020, and to tally all comments containing a word of interest related to infectious disease. The keywords searched were “chikungunya,” “Ebola,” “H1N1,” “MERS,” “SARS,” and “Zika.” They were selected a priori as common, unambiguous, and highly specific identifying references to 6 of the most publicized infectious disease epidemics of the past 2 decades. These were the first and only disease terms queried and analyzed.

The PushShift API search returns the total monthly incidence of each search term across all platform comments in the study period. It does not provide data on the overall number of monthly comments or total Reddit use. To control for changes in the total volume of Reddit posts over the study period, we also tallied the total number of comments containing each of 3 commonly used words not directly related to our subject matter: “to,” “a,” and “the.” After confirming that the relative use of all 3 words was substantially the same as each other (Multimedia Appendix 1), we selected the most frequently used word, “the,” as a proxy for total comment volume since the actual total comment volume is not possible to obtain. A large portion of comments will contain the word “the” and the proportion of comments containing “the” is unlikely to change substantially month-to-month, so it can be used as a reliable control for fluctuations in total comment volume over time. We determined the ratio of each infectious disease search term incidence divided by the incidence of “the” for each month of the study period, allowing for comparisons across study period months. For clarity and ease of display, these monthly ratios are expressed as the number of comments that include a given disease search term per 10,000,000 comments that include the word “the.”

For each search term, the monthly incidence ratios (search term comments per 10,000,000 “the” comments) from January 2018 through December 2019 were used to derive a pre-COVID mean incidence ratio as a baseline for comparison. Each individual monthly ratio from 2020, after COVID entered the public consciousness, was then compared to the corresponding search term’s pre-COVID mean using an unpaired 2-tailed *t* test. Since the analysis involved 12 independent comparisons for each search term (1 per month of 2020), we used the Bonferroni correction to set a significance cutoff of *P*=.004 (.05/12).

Google Trends data were obtained from the Google Trends site [16]. Data were collected for each of the 6 search terms from January 2018 through December 2020. Parameters selected were “worldwide,” “all categories,” and “web search.” The analysis for Google Trends was similar to the analysis for Reddit, with 2 methodologic exceptions: (1) Google Trends returns weekly rather than monthly figures and (2) the data need not be controlled for changes in total volume because they represent each term’s relative weekly popularity, normalized to the search popularity of the term’s most popular week. Google Trends assigns a value of 1 to 100 to each week in the search period, with 100 corresponding to the week of the search term’s greatest popularity in the period and all other weeks receiving values proportional to their popularity relative to the peak week. For example, a 5 would represent one twentieth of the relative search popularity compared to the week with the highest relative search popularity. Whereas the absolute incidence of each search term in Reddit comments must be normalized for total monthly volume as described in the preceding paragraph, no such correction is required for the Google Trends data, which is only available in the form of relative popularity scores, already normalized for total volume by default. After collecting this normalized Google relative popularity value for each week over the 3-year study, the mean value for each of the 6 infectious disease search terms was calculated. Weekly values for each week of 2020 were then compared with that term’s mean relative popularity using an unpaired 2-tailed *t* test with a significance cutoff of *P*=.00096 (using the Bonferroni correction of .05 / 52, given 1 comparison for each week of the year). Because Google Trends provides only relative data, 2 data sets with different “peak week” reference points cannot be validly compared, precluding the comparison of 2020 weekly popularity values to a mean 2018-2019 pre-COVID baseline; rather, the 2020 weekly values were compared to the mean relative popularity value of the entire 3-year study period.

### Ethical Considerations

As only publicly available, aggregated, anonymous, and nonidentifiable data were used, an institutional review board’s ethical review was not required.

## Results

**Figure 1:**
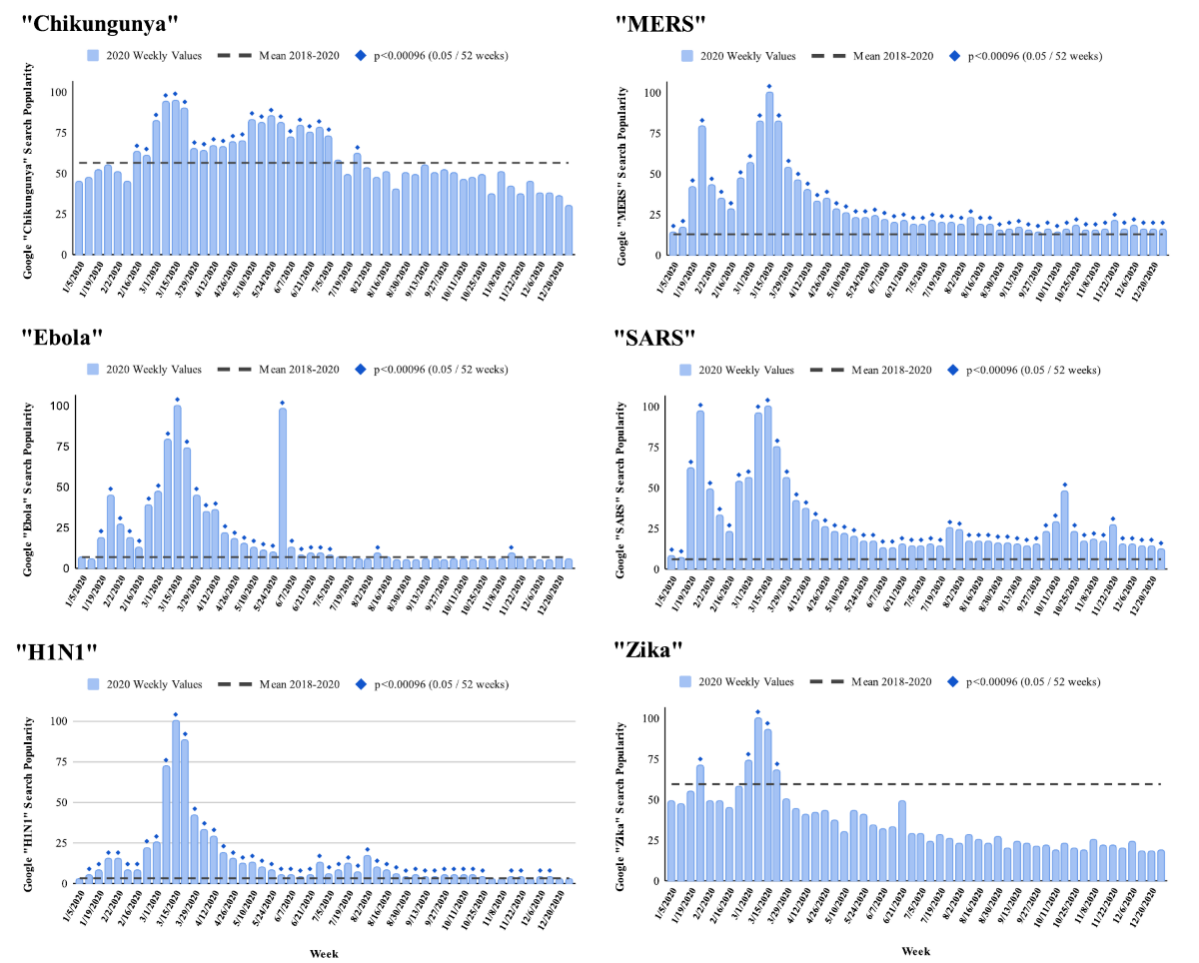
Google Search Trends for “chikungunya,” “Ebola,” “H1N1,” “MERS,” “SARS,” and “Zika.” Blue bars show the 2020 portion of the 2018-2020 relative weekly search volume distribution normalized to the week with the greatest search term popularity in the period, which is set to a value of 100. The dashed horizontal line represents the mean relative popularity across the 156 weeks of the study period, and statistically significant 2020 weekly deviations from that mean are indicated with a diamond.

Google searches and Reddit comments that included each of the 6 selected infectious disease search terms rose significantly in the first months of 2020, above the pre-COVID baseline usage of each term (raw data are provided in Multimedia Appendices 2 and 3). Google searches for “SARS” and “MERS” rose first, elevating above the pre-COVID baseline in the first week of 2020, peaking on the week of March 15, and remaining elevated for the entirety of the calendar year (*P*<.001, for each of the 52 weeks; Figure 1). The week of March 15, 2020, also marked the Google Trends peak for “Ebola,” “chikungunya,” and “H1N1,” each of which was elevated above the pre-COVID baseline by the week of February 16 through at least June 7, 2020. “Ebola” also had a second peak, narrow but nearly as high as the first, on the week of May 31, 2020, which corresponded to an announcement of an Ebola outbreak in the Democratic Republic of the Congo. Of the 6 search terms for which Google Trends data were evaluated, “Zika” had the highest pre-COVID average normalized relative popularity value, at 59.6. Its peak above this baseline was the narrowest of the 6 search terms, lasting only for the first 4 weeks of March 2020 and peaking the week of March 8, 2020. For that 4-week period of spring 2020, the relative search volume for all 6 diseases was elevated above the pre-COVID baseline to a highly significant degree (*P*<.00096 for all 24 values, corresponding to each of the 6 search terms through that 4-week period).

**Figure 2:**
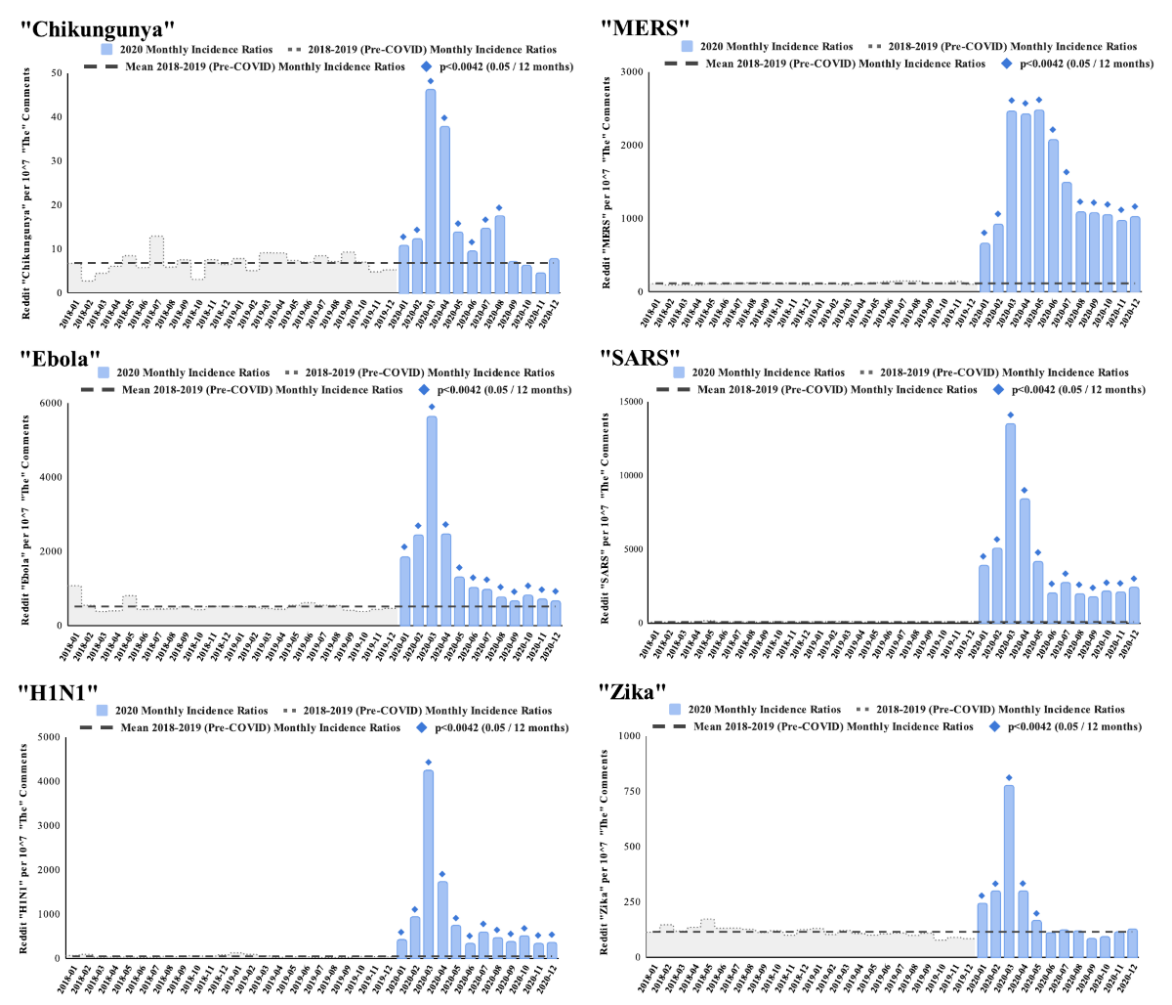
Reddit comments containing the terms “chikungunya,” “Ebola,” “H1N1,” “MERS,” “SARS,” or “Zika” per 10,000,000 comments with the word “the” as a proxy for total Reddit monthly comment volume. For each disease term, normalized comment volumes for each month in 2020 (blue bars) are compared with the pre-COVID baseline frequency expressed as a mean incidence ratio (term comments per 10,000,000 “the” comments) from the 24 pre-COVID months of 2018-2019 (dashed line, carried through the 2020 bars for ease of comparison). The monthly data for 2018 and 2019 are represented as a gray-stepped area curve. When the normalized monthly comment volume is close to 0, the gray curve may not be visible.

The proportion of Reddit comments containing the words “chikungunya,” “Ebola,” “H1N1,” “SARS,” and “Zika” each increased in January and February 2020 and then peaked in March 2020 (Figure 2). “MERS” showed a similar pattern, with an initial increase in January and February 2020, but comments peaked in May rather than March 2020. Reddit references to each of the 6 evaluated diseases remained elevated well above the pre-COVID baseline for at least the first 5 months of 2020 (*P<*.001) for each of the 30 monthly comparison points. “Zika” returned to its pre-COVID baseline in June 2020, followed by “chikungunya” in September 2020. References to “Ebola,” “H1N1,” “MERS,” and “SARS” remained elevated for the entirety of 2020. Although “chikungunya” peaked in March and the increase was statistically significant until September, the total search volume was low compared to the other 5 terms, and the pattern was less striking, likely due to overall lower levels of public awareness of this disease.

## Discussion

This study uses 2 large public data sets to assess changes in the degree of public interest in recent epidemic diseases after the outbreak of a new epidemic. For all 6 infectious disease search terms evaluated, interest increased after the first reports of COVID-19 and peaked within 3 to 5 months. This pattern was evident regardless of the causative pathogen classification, transmission method, clinical presentation, or geographic distribution of the prior epidemic diseases. The initial increases in January and February 2020 correspond to widespread news and publicity related to the initial outbreaks in China and Europe, while the March peaks correspond with increased spread to other parts of the world, including the United States. For “Ebola,” “H1N1,” “MERS,” and “SARS,” the increases persisted at least through the end of 2020.

The increased use of these infectious disease terms in both Google Trends and Reddit immediately following the outbreak of COVID-19 indicates elevated public interest in a wide variety of infectious diseases of epidemic potential at that time. The Reddit data in particular suggest a high degree of public engagement with these diseases because the words were used in active commentary and dialogue rather than simply as search terms. These data trends may indicate increased public awareness or consciousness of a wide variety of infectious diseases during the initial COVID surge period as individuals considered the surprising impact of the burgeoning epidemic. It seems likely that the persistent elevated use of some of these terms in both Google searches and Reddit comments even several months later is related to the COVID pandemic, considering the significant increased use compared to months preceding COVID. With the exception of the Ebola outbreak in 2020, we are not aware of any other epidemics, worldly events, or activities that would trigger increased use of these terms.

Our study is retrospective and observational and therefore cannot assert a causal relationship between COVID-19 and increased public interest in the 6 diseases evaluated. We do not have data to determine the extent to which the increased use of the infectious disease search terms can be attributed to individuals using them many times rather than many individuals each using them only once or a small number of times. The 6 search terms included for analysis were selected as common, unambiguous, and specific references to widely publicized epidemics in the 2 decades preceding COVID-19; they do not comprise a comprehensive set of the terms used to identify those 6 diseases in internet searches or Reddit comments, and the 6 epidemic diseases they refer to do not comprise a complete accounting of recent epidemics. However, the terms were selected a priori, and they do comprise the complete set of search terms evaluated for this study; that is, the fact that we derived strongly suggestive results with consistent patterns across each and every term evaluated without exception or exclusion increases the likelihood of replicability in other data sets, including future ones. Additional studies, including prospective investigations during future epidemics, will be useful to verify whether the patterns noted in this study recur reliably and to what extent they are generalizable. Fortunately, the barriers to future tests of replicability are minimal, given the free public availability of the data sources evaluated here.

We have less cause for confidence that our findings may be generalizable to noninfectious epidemics, but the digital tools used can indicate real-time public interest so readily that even hunches can be checked immediately at essentially no cost. For a variety of parties who could benefit from knowing when public engagement toward a given disease is rising, our data are certainly suggestive enough to check at the onset of a new epidemic disease using the tools described here.

The existence of predictable opportunities to more effectively target public health campaigns would be a silver lining amid the devastation of an epidemic, a chance to invest limited public health resources when they have the most potential for benefit. Guidelines for effective public health programs include effective timing as a key element [17]. Timing public health campaigns and research fundraising efforts to coincide with maximum target audience engagement can potentiate the impact of those efforts. We have demonstrated that for 6 distinct infectious causes of epidemics over the last 2 decades, public interest rose substantially and rapidly with the outbreak of COVID-19. Our data suggest that for at least several months afterward, the public may have been particularly receptive to dialogue on these topics. The tools used in this study, Google Trends and Reddit search, may be easily applied at low cost to examine public interest trends in real time to optimize public health education, patient advocacy, fundraising campaigns, and research investment.

## Appendix

Multimedia Appendix 1 Monthly incidence of Reddit comments containing each of 3 common English language words, showing a close correlation between all 3 over time.

Multimedia Appendix 2 Raw data for Google Trends relative popularity for each search term by week from January 2019 to December 2020. Selected parameters were “worldwide,” “all categories,” and “web search.”.

Multimedia Appendix 3 Raw data for the number of Reddit posts with each search term from January 2018 to December 2020.

## Abbreviations

MERS: Middle Eastern respiratory syndrome
SARS: severe acute respiratory syndrome

## Data Availability

All data generated or analyzed during this study are included in this published article and its supplementary information files.
