# Supplementary figures and images for: Reddit and Google Activity Related to Non-COVID Epidemic Diseases Surged at Start of COVID-19 Pandemic: Retrospective Study
Source: JMIR Form Res. 2023 Jul 6;7:e44603. doi: 10.2196/44603 (PMC10337364; doi:10.2196/44603)

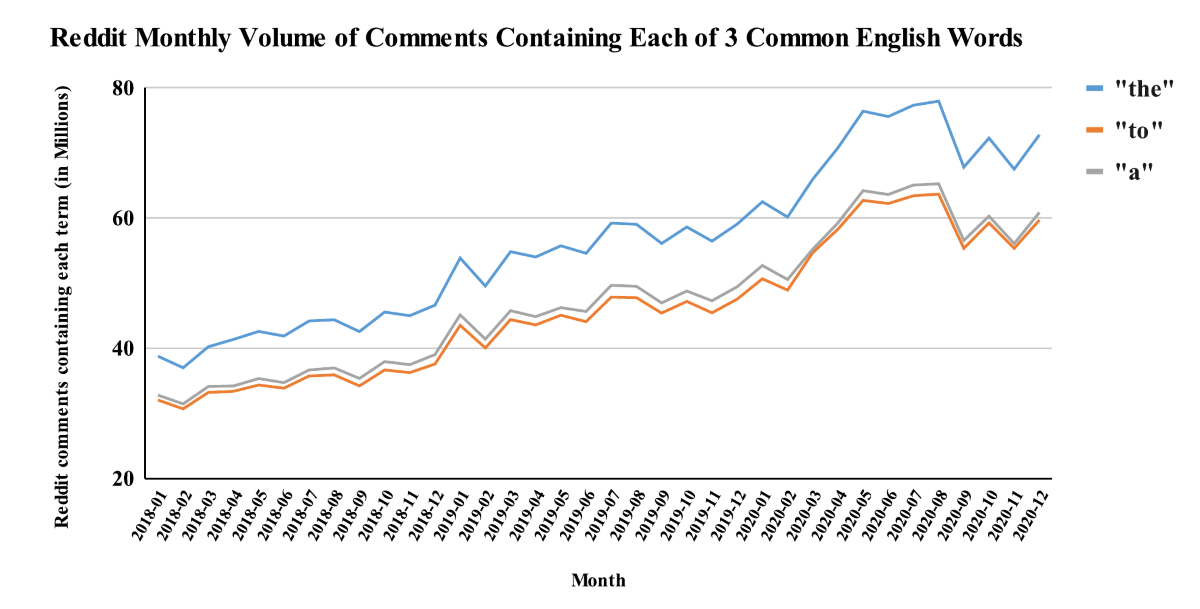

Supplement: Multimedia Appendix 1 [file formative_v7i1e44603_app1.png]
